# Multimorbidity patterns by health-related quality of life status in older adults: an association rules and network analysis utilizing the Korea National Health and Nutrition Examination Survey

**DOI:** 10.4178/epih.e2022113

**Published:** 2022-11-29

**Authors:** Thi-Ngoc Tran, Sanghee Lee, Chang-Mo Oh, Hyunsoon Cho

**Affiliations:** 1 Department of Cancer Control and Population Health, National Cancer Center Graduate School of Cancer Science and Policy, Goyang, Korea; 2 Department of Preventive Medicine, Kyung Hee University School of Medicine, Seoul, Korea; 3 Department of Cancer AI and Digital Health, National Cancer Center Graduate School of Cancer Science and Policy, Goyang, Korea; 4 Integrated Biostatistics Branch, Division of Cancer Data Science, Research Institute, National Cancer Center, Goyang, Korea

**Keywords:** Multimorbidity, Health-related quality of life, Network analysis, Association rule, Older adults

## Abstract

**OBJECTIVES:**

Improved life expectancy has increased the prevalence of older adults living with multimorbidity which likely deteriorates their health-related quality of life (HRQoL). However, relatively little is known about patterns and the relationships of multimorbidity by HRQoL status in older adults.

**METHODS:**

Individuals aged 65 or older from the Korea National Health and Nutrition Examination Survey V-VII (2010-2018) were analyzed. HRQoL was assessed by the EuroQoL-5 dimensions questionnaire and categorized as poor, normal, or good. The impact of multimorbidity on HRQoL was evaluated using logistic regression. The patterns and inter-relationships between multimorbidity, stratified by HRQoL groups, were analyzed using the association rules and network analysis approach.

**RESULTS:**

Multimorbidity was significantly associated with poor HRQoL (3 or more diseases vs. none; adjusted odds ratio, 2.70; 95% confidence interval, 2.10 to 3.46). Hypertension, arthritis, hyperlipidemia, and diabetes were the most prevalent diseases across all HRQoL groups. Complex interrelationships of morbidities, higher prevalence, and node strengths in all diseases were observed in the poor HRQoL group, particularly for arthritis, depression, and stroke, compared to other groups (1.5-3.0 times higher, p<0.05 for all). Apart from hypertension, arthritis and hyperlipidemia had a higher prevalence and stronger connections with other diseases in females, whereas this was the case for diabetes and stroke in males with poor HRQoL.

**CONCLUSIONS:**

Multimorbidity patterns formed complicatedly inter-correlated disease networks in the poor HRQoL group with differences according to sex. These findings enhance the understanding of multimorbidity connections and provide information on the healthcare needs of older adults, especially those with poor HRQoL.

## GRAPHICAL ABSTRACT

[Fig f3-epih-44-e2022113]

## INTRODUCTION

The growing aging population has led to an increase in older adults living with multimorbidity, worsening their health-related quality of life (HRQoL). Multimorbidity is defined as the coexistence of 2 or more diseases in the same individual [1-3], and older age is substantially correlated with multimorbidity [2,4-7]. For example, in Korea, a rapidly aging society, around one-third of people over 50 years had 2 or more coexisting diseases [4]. Multimorbidity reduces the likelihood of receiving preventive care and increases medical services demands, healthcare costs, polypharmacy, and consequently impairs physical functions and quality of life (QoL) and survival in older adults [8-12].

HRQoL is a multidimensional concept that provides a wideranging perspective on health. As a comprehensive appraisal of disease burden, HRQoL incorporates assessments of an individual’s ability to function in life’s physical, mental, and social domains. Socioeconomic status, physical activity or sleeping status, and multimorbidity impact an individual’s HRQoL [4,13-15]. A recent study in Korea showed that higher morbidity was associated with significantly lower HRQoL [4]. Other studies with older adults with a history of stroke revealed that impaired HRQoL was associated with the number and type of strokes [16]. However, these studies solely focused on a single disease or 2 diseases and thus did not address the correlations and complex interrelationships of the morbidities. Divergences in the disease network that may exist according to HRQoL status in older adults remain unclear.

The effects of morbidities on HRQoL are not separate, since these morbidities coexist in patterns of multimorbidity, especially in older adults [17]. Previous studies have shown that the impact of multimorbidity patterns on declining HRQoL was different across clusters of multimorbidity and types of HRQoL components [17-19]. Conducting more research to discover the inter-correlations between morbidities would help identify the high-risk population and measure the effect of morbidities on HRQoL more accurately [17,19,20].

In recent years, association rules and multimorbidity network analyses have been applied to explore the connections between diseases [21-25]. Association rules have been a popular method for assessing the association between items applied to a set of 2 diseases to identify the prevalence and relationship of diseases based on 3 primary measurements: support, confidence, and lift. Network graphics based on association rules help to identify highly connected diseases in multimorbidity networks and show the connections of each disease with all others, resulting in a representation of the pattern of multimorbidity (i.e., how diseases tend to cluster together or co-occur) [6]. One such study suggested that the multimorbidity pattern differed by sex and people under and over 65 [22]. Another study among 50 older Irish adults identified a more highly connected multimorbidity network in females than in males and strong associations between arthritis, osteoporosis, and thyroid issues [23]. However, those studies did not investigate multimorbidity networks across the different statuses of HRQoL and quantify its diverse impact.

This study aimed to investigate complex patterns and inter-relationships of multimorbidity in older adults by HRQoL status using association rules and network analysis. We specifically focused on evaluating how multimorbidity status is associated and correlated with impaired HRQoL in people aged 65 years and over.

## MATERIALS AND METHODS

### Data source

Data were obtained from the Korea National Health and Nutrition Examination Survey (KNHANES) V-VII (2010-2018), conducted by the Korean Centers for Disease Control and Prevention (renamed in 2020 as the Korea Disease Control and Prevention Agency). The KNHANES is an annual nationwide cross-sectional health survey that collects information on health status, socioeconomic status, health determinants, QoL, and healthcare utilization [26]. Participants aged 65 or over were our research’s target population [2]. Participants with missing HRQoL measures (EuroQoL-5 dimensions-3 level [EQ-5D-3L] index, 10.9%) were excluded.

### Measurements

The 15 chronic diseases consistently measured in the 3 survey periods (V-VII) were selected and used to define morbidity in this study, including hypertension, arthritis, asthma, stroke, angina pectoris, myocardial infarction, diabetes, thyroid, renal failure, hyperlipidemia, liver disease, depression, tuberculosis, chronic kidney disease (CKD), and cancer. All measured cancer types, including stomach, liver, colon, breast, cervix, lung, thyroid cancer, and others, were combined into one variable, called “cancer.” The “liver disease” variable was calculated based on hepatitis B, hepatitis C, and liver cirrhosis. Except for CKD, the presence of a chronic disease (X) was examined based on the question: “Have you ever been diagnosed with disease X by a doctor?” CKD was defined as an estimated glomerular filtration rate of less than 60 mL/min/1.73 m^2^, calculated from the creatinine level in the health examination [27].

HRQoL was measured using the EQ-5D-3L questionaire questionnaire, one of the most widely used instruments to measure HRQoL, and it has been included in the KNHANES since 2005 [26]. Based on the responses to these questions, the EQ-5D index score was calculated using the Korean value set [28]. The EQ-5D index ranged from -0.17 to 1.00, with 1.00 indicating the best health. The lowest 5% of the EQ-5D index of the total adult KNHANES population (EQ-5D index score ≤ 0.721) was defined as the poor HRQoL group [15]. Then, the EQ-5D index was categorized into 3 groups: poor HRQoL (EQ-5D≤ 0.721), normal HRQoL (0.721< EQ-5D< 1.000), and good HRQoL (EQ-5D= 1.000) (Supplementary Material 1).

We included socio-demographic factors (age, sex, marital status, income, working status, and education) and health determinants (body mass index [BMI], smoking, high-frequency drinking status, walking, sleeping status, and stress) in the analysis. Further details of the variables can be found in Supplementary Material 2.

### Statistical analysis

Descriptive statistics were calculated to examine the characteristics of the older population stratified by HRQoL groups. Multivariable logistic regression analysis was used to evaluate the associations of poor HRQoL with multimorbidity status, adjusting for socio-demographic and health determinants. The complex survey design and weights were considered in the analysis. SAS version 9.4 (SAS Institute Inc., Cary, NC, USA) were used.

Network analysis using association rules was performed to identify the prevalence and interrelationship of diseases based on the three primary measurements: support, confidence, and lift [22,23,25,29]. Support represents the prevalence of the co-occurrence of 2 diseases (e.g., X and Y), denoted by *P(X,Y)*. Confidence is defined by the conditional probability of disease Y among people who have disease X, denoted by *P(Y|X)*. Lift is calculated as the observed to expected ratio and represents the degree of cooccurrence between *X* and *Y*, defined by $\frac{P\left(X,Y\right)}{P\left(X\right)\times P\left(Y\right)}$[22]. A lift value larger than 1 indicates that 2 diseases are more likely to cooccur. If the lift value equals 1, there is no association between them. Network illustration was conducted based on the support values to better visualize the interactions of multimorbidity in each HRQoL group [22-25]. A network graph consists of nodes (diseases) that are connected by edges (a straight line). The size of a node is proportional to the prevalence of the specific diseases, and the thickness of the edge connecting any 2 diseases in the network is the support value. In addition, the node strength was calculated based on the support value to assess how strongly directed the connection of a node was with other nodes in the network [30]. The color of the nodes in the network represents the node strength. A higher node strength value (darker the color) represents a stronger connection with other nodes. A heatmap graph was used to show all the lift values of association rules. All analyses were performed using the *arules, igraph*, and *ggplot* packages in R version 4.0.4 [30-32]. Details of the statistical analyses are shown in Supplementary Material 2.

### Ethics statement

This study used publicly available secondary de-identified data. The institutional review board (IRB) review exemption was approved by the IRB of the National Cancer Center of Korea.

## RESULTS

### Characteristics of the study population

Of the 12,657 participants aged 65 and older, 67.0% were unemployed, 62.1% had an elementary education, 19.6% experienced stress, 30.4% had 3 or more diseases, and 14.2% reported having poor HRQoL (Table 1). The data shows that 74.6% of those in the poor HRQoL group, 63.2% in the normal HRQoL group, and 45.4% in the good HRQoL group were female. Among people with poor HRQoL, 45.1% had 3 or more diseases, compared to 34.4% and 22.1% in the normal and good HRQoL groups, respectively.

### Impact of multimorbidity status on health-related quality of life

The odds ratios of reporting poor HRQoL by multimorbidity status adjusted by age, sex, marital status, income, education, working status, drinking and smoking status, walking, BMI, and stress are presented in Table 2. Multimorbidity status was significantly associated with impaired HRQoL. People who had 2 diseases and those who had 3 or more diseases were approximately 2 times (adjusted odds ratio [aOR], 1.88; 95% confidence interval [CI], 1.46 to 2.42) and 3 times (aOR, 2.70; 95% CI, 2.10 to 3.46) more likely to have poor HRQoL, respectively, than those without any diseases.

### Patterns and interrelationships of multimorbidity in the divergence of health-related quality of life status

**Figure 1.**
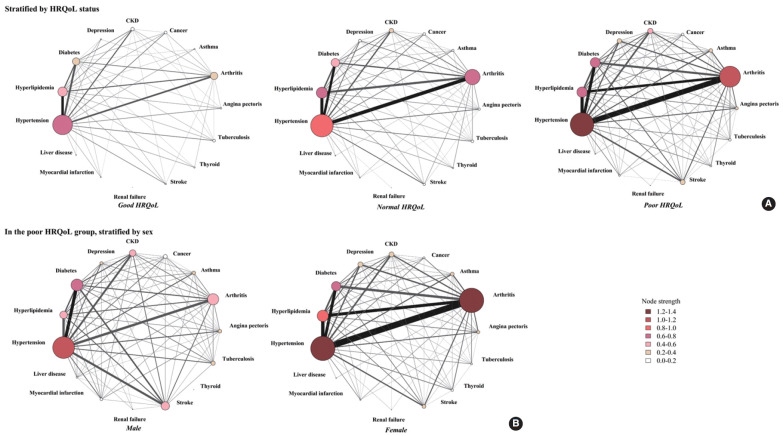
Network analysis of patterns and interrelationships of multimorbidity. (A) Multimorbidity patterns stratified by HRQoL status (good HRQoL, normal HRQoL, poor HRQoL); (B) Multimorbidity patterns in the poor HRQoL group, stratified by sex (male, female). The figure shows the connections among 15 diseases. The size of nodes represents the prevalence of diseases; a larger size means a higher prevalence. The color of the nodes represents the node strength of each node; a darker color means more connections of a node with other nodes. The thickness of the edges is the proportion to the number of people who had both diseases, which means that 2 diseases with thicker edges had a higher proportion of occurring together. HRQoL, health-related quality of life; CKD, chronic kidney disease.

The network of multimorbidity in each HRQoL status was visualized (Figure 1A) based on the support values in the association rules results (Supplementary Material 3), the prevalence, and node strength (Supplementary Material 4). Generally, the network of people with poor HRQoL was more complicated and had stronger connections between diseases. The most prevalent diseases in the total population, across all HRQoL groups, were hypertension (54.5%), arthritis (32.0%), hyperlipidemia (26.0%), and diabetes (20.8%). Meanwhile, the prevalence of arthritis, stroke, depression, and asthma in the poor HRQoL group was about 1.5 times higher than that in the normal HRQoL group and 3.0 times higher than that in the good HRQoL group (all p-values< 0.05) (Figure 1A, Supplementary Material 4). Moreover, the support values of the co-occurrence of pairs of disease in the poor HRQoL group were higher than in the normal and good HRQoL groups: arthritis and hypertension (35.1% in the poor HRQoL group vs. 23.4% in the normal HRQoL group and 10.5% in the good HRQoL group), arthritis and hyperlipidemia (16.4% in the poor HRQoL group vs. 13.8% in the normal HRQoL group and 5.8% in the good HRQoL group), hypertension and stroke (9.3% in the poor HRQoL group vs. 4.6% in the normal HRQoL group and 2.8% in the good HRQoL group), and arthritis and depression (7.3% in the poor HRQoL group vs. 3.5% in the normal HRQoL group and 0.9% in the good HRQoL group).

The results for node strength showed that hypertension, arthritis, hyperlipidemia, and diabetes had the most substantial connections with other diseases in all HRQoL groups (Figure 1A, Supplementary Material 4). These 4 diseases had the largest connections to each other in the network, and the level increased considerably from the good HRQoL group to the poor HRQoL group. The most vital disease in the network was hypertension, as indicated by the highest node strength value, regardless of the HRQoL status (1.30 in the poor HRQoL group, 0.98 in the normal HRQoL group, and 0.66 in the good HRQoL group). However, the role of arthritis significantly increased as HRQoL decreased; for instance, the node strength of arthritis in the good HRQoL group was 0.30, and it significantly increased to 0.69 in the normal HRQoL group and 1.09 in the poor HRQoL group. The node strengths of hyperlipidemia, diabetes, CKD, depression, and stroke were also much higher in the poor HRQoL group than in others.

The results of the confidence values in the association rules (Supplementary Material 3) illustrated that more than 70% of people with CKD or stroke also reported hypertension regardless of HRQoL status, which means that hypertension was more likely to co-occur with another disease.

**Figure 2.**
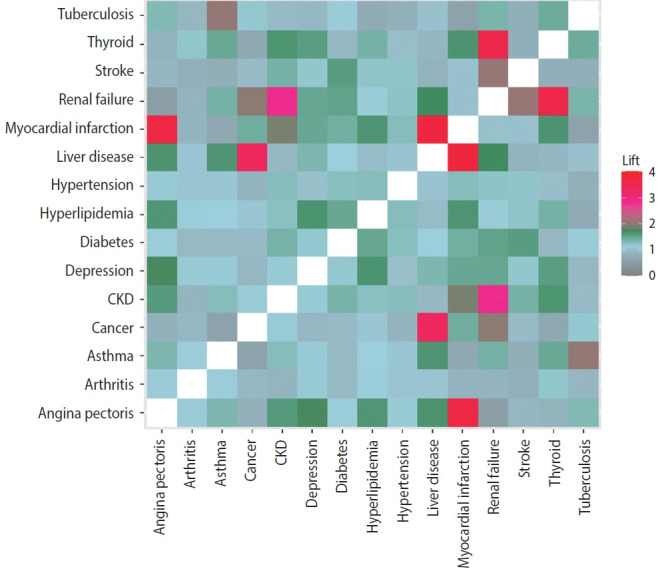
Heatmap of lift values for interrelationships of multiple morbidities in the poor HRQoL group. Each grid represents the lift value, which means the degree of co-occurrence between 2 diseases on the x- and y-axes. A lift value larger than 1 indicates that 2 diseases are more likely to co-occur: the higher the lift, the higher chance of co-occurrence. HRQoL, health-related quality of life; CKD, chronic kidney disease.

The lift measurements for the co-occurrence of 2 diseases in the poor HRQoL group were displayed on a heatmap (Figure 2). Many sets of 2 diseases co-occurred more frequently than would be expected by random chance in the poor HRQoL group. For example, renal failure and thyroid issues (lift value 3.6), cancer and liver disease (lift value 3.4), stroke and renal failure (lift value 2.0), CKD and myocardial infarction (lift value 1.9), and CKD and thyroid issues (lift value 1.7) co-occurred more frequently than would be expected. Two respiratory system diseases (tuberculosis and asthma) in the poor HRQoL group were also shown to accompany each other twice more frequently than would be expected (lift value 2.0). Some high-prevalence diseases, such as hyperlipidemia and diabetes, appeared about 1.5 times more than expected by random chance. Each line in the heat map shows the degree of co-occurrence of a disease with all other diseases. Hypertension and arthritis mostly occurred with other diseases at 1.1-1.2 times higher frequencies than would be expected by random chance. Two diseases of the kidney, CKD and renal failure, appeared together at a higher chance than chance (lift value 2.8) and shared the same pattern of co-occurrence with stroke and thyroid (all lift values > 1.4). Angina pectoris, a cardiovascular disease, was reported to co-occur with myocardial infarction, hyperlipidemia, depression, liver disease, and CKD more than would be expected by random chance (all lift values > 1.5).

### Patterns and interrelationships of multimorbidity in each health-related quality of life group by sex

The prevalence and node strength of multimorbidity were assessed by sex and HRQoL status (Table 3). Hypertension was consistently prevalent in males and females regardless of their HRQoL status. In males, apart from hypertension, diabetes was the most critical multimorbidity, especially in the poor HRQoL group in terms of prevalence (18.9% in the good HRQoL group, 22.5% in the normal HRQoL group, and 30.4% in the poor HRQoL group) and the node strength (0.31 in the good HRQoL group, 0.42 in the normal HRQoL group, and 0.54 in the poor HRQoL group). However, in females, the corresponding disease was arthritis, in terms of prevalence (31.4% in good, 50.8% in the normal HRQoL group and 62.8% in the poor HRQoL group) and the node strength (0.51 in the good HRQoL group, 0.90 in the normal HRQoL group and 1.27 in the poor HRQoL group). This divergent multimorbidity pattern between male and female existed in each HRQoL group and was observed clearly in the poor HRQoL group.

Further, a network analysis of poor HRQoL was performed (Figure 1B) based on the prevalence, node strength results (Table 3), and the support values of association rule results (Table 4) to better visualize the pattern of multimorbidity. Females with poor HRQoL had higher prevalence rates and stronger associations between morbidities than males. Overall, in males, hypertension (node strength 1.1), diabetes (node strength 0.7), arthritis (node strength 0.6), hyperlipidemia (node strength 0.5), stroke (node strength 0.5), and CKD (node strength 0.4) were the diseases that had the most connections with others. In females, the order was different: hypertension (node strength 1.4), arthritis (node strength 1.3), hyperlipidemia (node strength 0.8), diabetes (node strength 0.7), CKD (node strength 0.4), and depression (node strength 0.4). A higher node strength of morbidities, especially arthritis, hyperlipidemia, and depression, was observed in females than in males. By contrast, diabetes and stroke occurred more frequently with high node strength in males (Table 3). In addition, the support values of the co-occurrence of pairs of disease in females were much higher than in males, as exemplified by arthritis and hypertension (41.6% in female vs. 15.8% in male), arthritis and hyperlipidemia (20.1% in female vs. 5.5% in male), and arthritis and diabetes (15.8% in female vs. 6.3% in male) (Table 4 and Figure 1B). In contrast, the support values of the co-occurrence of stroke and hypertension (13.8% in male vs. 7.8% in female) and stroke and diabetes (9.4% in male vs. 3.7% in female) were higher in the multimorbidity network of male than in that of female (Table 4 and Figure 1B). The association rules (Table 4) also showed that even though the proportion of co-occurrence of hypertension and diabetes was slightly higher in male than in female (support value 20.8% in male vs. 19.1% in female), the multimorbidity pattern was different according to the confidence value. Among males who had diabetes, 68.3% had hypertension, while among females who had diabetes, 79.3% had hypertension. Additionally, among males with hypertension, 37.3% had diabetes, while only 30.5% of females who had hypertension also had diabetes. These 2 diseases occurred more frequently in females than males (lift value 1.3 in female vs. 1.2 in male).

## DISCUSSION

In this study, we applied network analysis methods to illustrate the complex interrelationships of multimorbidity according to HRQoL status in older adults. We utilized association rules to evaluate the interrelationships and visualized them with network-type plots. This approach uncovered diverse multimorbidity patterns in older adults with different HRQoL statuses.

Multimorbidity was associated with poor HRQoL after adjusting for health determinants and socio-demographic characteristics. Furthermore, the association rules and network analysis approach illustrated a higher prevalence of arthritis, depression, stroke, and stronger correlations among morbidities in the poor HRQoL group, especially female participants. Our findings expand the understanding of multimorbidity patterns in older adults and guide health care providers to consider disease in older adults more comprehensively instead of considering only a single disease at a time. Hence, these results imply the necessity of assessing the integrated impact of these morbidities on HRQoL and providing appropriate services to improve older adults’ health status.

Our study showed that the multimorbidity status was exacerbated, and the number of coexisting morbidities significantly increased as HRQoL deteriorated in individuals aged 65 years or older. Previous studies have also reported similar results in China, Korea, and other Asian countries [4,5,13]. Consistent with previous studies [22,23,33], our network results indicated that hypertension had stronger connections with hyperlipidemia and diabetes than other pairs of morbidities in older adults. Our study also showed similar results to another study among Koreans aged 50 and older that classified hypertension, hyperlipidemia, diabetes, and stroke as a single latent class of multimorbidity [34]. In addition, our study emphasized that these connections were stronger in the poor HRQoL group, which has not been discovered in previous studies. The substantial overlap between hypertension, diabetes, and hyperlipidemia in etiology and disease mechanisms might have resulted in this phenomenon. For instance, diabetes and hypertension have been proven to share common pathways such as oxidative stress, insulin resistance, and metabolic syndrome, which interact and subsequently influence the prevalence of these 2 diseases [23]. Further, this study highlights that arthritis had connections with all remaining diseases. Lower HRQoL was associated with stronger links, especially for the association rules of arthritis with hypertension and depression. Several studies have shared similar results regarding the combination of arthritis with other diseases [4,35,36].

The results of lift values in the association rules analysis and heatmap provided probabilities of combinations of morbidities that occur more frequently than expected by random chance. For instance, thyroid issues and renal failure and thyroid issues and CKD co-occurred about 4 times and 2 times more often than would be expected, respectively, meaning that thyroid issues and renal diseases were dependent on each other. A recent review showed that hypothyroidism was a risk factor for developing renal disease; however, further studies are needed to understand the causal association between thyroid and renal diseases [37]. In addition, patients with CKD had myocardial infarction about 2 times more often than would be expected by chance in our study, which is consistent with a recent study about cardiovascular disease in the CKD population. The pathophysiology could involve CKDassociated mediators or hemodynamic alterations resulting in cardiovascular calcification and cardiac damage [38]. These results might help in clinical practice if health care providers recognize the risk of the second disease or co-occurrence of morbidities in older patients.

The prevalence and node strength of multimorbidity showed different patterns between male and female, especially in those with poor HRQoL. The female participants with poor HRQoL had a more complex network and a higher prevalence of diseases, especially arthritis, hyperlipidemia, and depression. Although the mechanism behind this finding is unclear, it is likely that physical inactivity, employment conditions, and stress are associated with the development of these diseases in females with poor HRQoL [39,40]. The network results also showed that female with poor HRQoL had a stronger connection between arthritis and depression, consistent with a previous study [36]. Another study among middle-aged Koreans also pointed out that females were more likely to develop arthritis, asthma, allergy, depression, and thyroid disease than males [34]. Our study also showed the interaction of arthritis with hypertension and depression; apart from that, our network results further showed that arthritis co-occurred with diabetes, CKD, renal failure, and angina pectoris more in females than males, which was less mentioned in the previous research. In contrast, diabetes and stroke had a higher prevalence and stronger associations in male with poor HRQoL than in female. Previous studies have shown that male sex is one of the risk factors for developing stroke in people with type 2 diabetes [41,42]. These sex-dependent multimorbidity patterns may inform health care practices by providing support for utilizing different strategies for males and females to achieve better management of multimorbidity and improve HRQoL.

Some limitations should be considered when interpreting the results of our study. Firstly, we conducted the association rules and network analysis based on a cross-sectional design, which did not allow us to establish causal relationships among multimorbidity patterns. Identifying causal relationships among the inter-related diseases in older adults would be left as a future possibility. Second, the KNHANES data only contained the most common disease types, and people with severe conditions in the hospital were likely to be excluded at the time of the survey. This may have resulted in underestimating the relationships between multimorbidity in the poor HRQoL group. Finally, we analyzed HRQoL using the EQ-5D index, which has national social values derived from the Korean population [28]. The EQ-5D index has a skewed nature and a high proportion of perfect scores resulting in high mean scores, which may cause a lack of discrimination when analyzing HRQoL [43]. Given this phenomenon, we categorized the lowest 5% of EQ-5D utility scores as the poor HRQoL group, the highest EQ-5D score (EQ-5D= 1) as the good HRQoL group, and the rest as the normal HRQoL group [15]. We suggest that this approach might allow a better discrimination of HRQoL status.

In conclusion, our findings based on the network method expanded the understanding of multimorbidity patterns in older adults with divergent HRQoL statuses and could serve to improve appropriate health service provision. The approach of association rules and network analysis provided an in-depth assessment of complex interrelationships between multiple morbidities and emphasized the connections of vital diseases with others. Hypertension, arthritis, hyperlipidemia, and diabetes were the most common morbidities, regardless of HRQoL status; however, these diseases were more frequent in people with poor HRQoL. Apart from hypertension, as HRQoL deteriorated, the network results showed that the prevalence and node strength of arthritis, depression, and stroke increased considerably. Furthermore, the multimorbidity networks differed by sex, especially in people with poor HRQoL, with higher prevalence and interactions of arthritis and hyperlipidemia in females; and diabetes and stroke in males. Hence, strategies aimed at health care provision for older adults should assess specific diseases, the connections between multiple morbidities in individuals, and sex differences to obtain effective results.

## Figures and Tables

**Figure f3-epih-44-e2022113:**
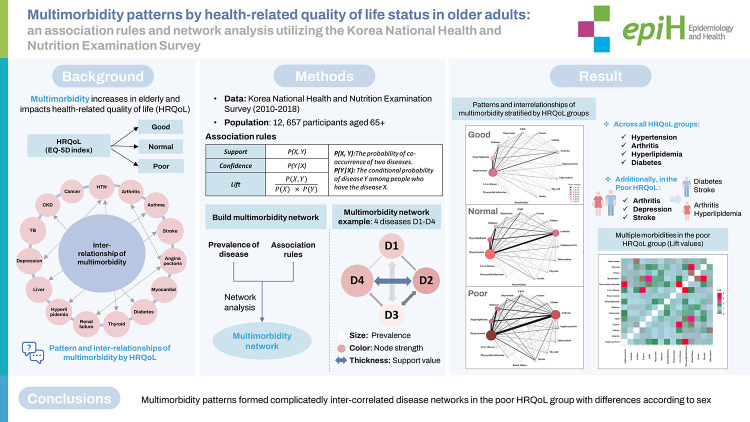


**Table 1.** Characteristics of the older population stratified by HRQoL groups (n=12,657)^1^

Characteristics		Total	HRQoL		
		(n=12,657)	Good (n=5,677)	Normal (n=5,177)	Poor (n=1,803)
Age					
	65-69	4,234 (33.4)	2,221 (39.1)	1,637 (31.6)	376 (20.8)
	70-74	3,824 (30.2)	1,783 (31.4)	1,555 (30.0)	486 (26.9)
	75-79	2,840 (22.4)	1,123 (19.8)	1,210 (23.4)	507 (28.1)
	≥80	1,759 (13.9)	550 (9.7)	775 (15.0)	434 (24.1)
Sex					
	Male	5,462 (43.2)	3,098 (54.6)	1,907 (36.8)	457 (25.3)
	Female	7,195 (56.8)	2,579 (45.4)	3,270 (63.2)	1,346 (74.6)
Marital status					
	Married	8,369 (66.1)	4,211 (74.2)	3,250 (62.8)	908 (50.4)
	Others^2^	4,215 (33.3)	1,435 (25.3)	1,896 (36.6)	884 (49.0)
Income					
	Low	3,126 (24.7)	1,183 (20.8)	1,345 (26.0)	598 (33.2)
	Middle	6,303 (49.8)	2,817 (49.6)	2,596 (50.1)	890 (49.4)
	High	3,124 (24.7)	1,628 (28.7)	1,202 (23.2)	294 (16.3)
Working status					
	Employed	4,181 (33.0)	2,207 (38.9)	1,612 (31.1)	362 (20.1)
	Unemployed	8,476 (67.0)	3,470 (61.1)	3,565 (68.9)	1,441 (79.9)
Education					
	Elementary or below	7,864 (62.1)	2,923 (51.5)	3,472 (67.1)	1,469 (81.5)
	Secondary or high school	3,664 (28.9)	2,020 (35.6)	1,368 (26.4)	276 (15.3)
	College or higher	1,050 (8.3)	711 (12.5)	305 (5.9)	34 (1.9)
Body mass index					
	Underweight	423 (3.3)	178 (3.1)	155 (3.0)	90 (5.0)
	Normal	4,465 (35.3)	2,148 (37.8)	1,736 (33.5)	581 (32.2)
	Overweight	3,242 (25.6)	1,530 (26.9)	1,321 (25.5)	391 (21.7)
	Obesity	4,475 (35.3)	1,803 (31.7)	1,947 (37.6)	725 (40.2)
Smoking status					
	No	11,150 (88.1)	4,963 (87.4)	4,597 (88.8)	1,590 (88.2)
	Yes	1,345 (10.6)	661 (11.6)	526 (10.2)	158 (8.8)
High-frequency drinking status					
	No	12,051 (95.2)	5,387 (94.9)	4,940 (95.4)	1,724 (95.6)
	Yes	455 (3.6)	243 (4.3)	185 (3.6)	27 (1.5)
Walking					
	No	7,881 (62.3)	3,088 (54.4)	3,400 (65.7)	1,393 (77.3)
	Yes	4,628 (36.6)	2,537 (44.7)	1,719 (33.2)	372 (20.6)
Sleeping status					
	Short	5,596 (44.2)	2,376 (41.8)	2,387 (46.1)	833 (46.2)
	Optimal	6,145 (48.5)	2,961 (52.1)	2,428 (46.9)	756 (41.9)
	Long	712 (5.6)	272 (4.8)	285 (5.5)	155 (8.6)
Stress					
	No	10,000 (79.0)	4,988 (87.9)	3,970 (76.7)	1,042 (57.8)
	Yes	2,482 (19.6)	634 (11.2)	1,147 (22.1)	701 (38.9)
Multimorbidity status (disease)					
	No	2,126 (16.8)	1,297 (22.8)	686 (13.2)	143 (7.9)
	1	3,436 (27.1)	1,756 (30.9)	1,310 (25.3)	370 (20.5)
	2	3,242 (25.6)	1,366 (24.0)	1,399 (27.0)	477 (26.4)
	≥3	3,853 (30.4)	1,258 (22.1)	1,782 (34.4)	813 (45.1)

Values are presented as number (%); Percentages are rounded to the one decimal places. Due to the missing values in marital status (73, 0.6%), income (104, 0.8%), education (79, 0.6%), BMI (52, 0.4%) smoking (162, 1.3%), drinking (151, 1.2%), walking (148, 1.2%), sleeping status (204, 1.6%) and stress (175, 1.4%) variables, the total percentage is less than 100% in some categories. HRQoL, health-related quality of life.

1 The distribution of all variables was significantly different across groups (p<0.001, except smoking status p=0.002).

2 Others included people who were single; married, not living together; married, husband/wife passed away; or divorced.

**Table 2.** Logistic regression model for the association between poor HRQoL and multimorbidity (n=12,657)

Variables		Univariate model	Multivariable model^1^
		OR (95% CI)	aOR (95% CI)
Age			
	65-69	1.00 (reference).	1.00 (reference)
	70-74	1.44 (1.21, 1.71)^***^	1.19 (0.99, 1.43)
	75-79	2.17 (1.83, 2.58)^***^	1.43 (1.17, 1.74)^***^
	≥80	3.45 (2.88, 4.14)^***^	2.08 (1.66, 2.61)^***^
Sex			
	Male	1.00 (reference)	1.00 (reference)
	Female	2.70 (2.36, 3.09)^***^	1.23 (1.02, 1.49)^*^
Marital status			
	Married	1.00 (reference)	1.00 (reference)
	Others^2^	2.34 (2.07, 2.64)^***^	1.34 (1.15, 1.57)^***^
Income			
	Low	2.21 (1.84, 2.65)^***^	1.69 (1.38, 2.07)^***^
	Middle	1.57 (1.34, 1.85)^***^	1.45 (1.21, 1.72)^***^
	High	1.00 (reference)	1.00 (reference)
Working status			
	Employed	1.00 (reference)	1.00 (reference)
	Unemployed	2.09 (1.80, 2.42)^***^	1.56 (1.33, 1.83)^***^
Education			
	Elementary or below	7.59 (5.13, 11.24)^***^	3.73 (2.40, 5.80)^***^
	Secondary or high school	2.55 (1.69, 3.85)^***^	2.07 (1.33, 3.23)^*^
	College or higher	1.00 (reference)	1.00 (reference)
BMI			
	Underweight	2.09 (1.58, 2.77)^***^	1.84 (1.34, 2.52)^*^
	Normal	1.00 (reference)	1.00 (reference)
	Overweight	0.88 (0.75, 1.03)	0.90 (0.75, 1.09)
	Obesity	1.38 (1.20, 1.58)^***^	1.21 (1.04, 1.43)^*^
Smoking status			
	No	1.00 (reference)	1.00 (reference)
	Yes	0.80 (0.65, 0.98)^*^	1.17 (0.92, 1.50)
High-frequency drinking status			
	No	1.00 (reference)	1.00 (reference)
	Yes	0.37 (0.24, 0.59)^*^	0.57 (0.34, 0.93)^*^
Walking			
	No	2.56 (2.23, 2.94)^***^	1.86 (1.61, 2.17)^***^
	Yes	1.00 (reference)	1.00 (reference)
Sleeping status			
	Short	1.28 (1.12, 1.46)^***^	1.15 (1.00, 1.33)
	Optimal	1.00 (reference)	1.00 (reference)
	Long	1.95 (1.56, 2.45)^***^	1.70 (1.33, 2.17)^***^
Stress			
	No	1.00 (reference)	1.00 (reference)
	Yes	3.21 (2.81, 3.67)^***^	2.71 (2.34, 3.13)^***^
Multimorbidity status (disease)			
	No	1.00 (reference)	1.00 (reference)
	1	1.59 (1.27, 1.99)^***^	1.47 (1.14, 1.89)^*^
	2	2.31 (1.84, 2.90)^***^	1.88 (1.46, 2.42)^***^
	≥3	3.45 (2.75, 4.33)^***^	2.70 (2.10, 3.46)^***^

HRQoL, health-related quality of life; OR, odds ratio; aOR, adjusted odds ratio; CI, confidence interval; BMI, body mass index.

1 The multivariable model was adjusted for age, sex, marital status, income, working status, education, BMI, high-frequency drinking status, walking, sleeping status, stress, and multimorbidity status.

2 Others included single; married, not living together; married, husband/wife passed away; or divorced.

* p<0.05,

*** p<0.001.

**Table 3.** Prevalence of diseases and node strength of the multimorbidity network in each HRQoL group, stratified by sex

Type of diseases		Prevalence of diseases, n (%)			Node strength of multimorbidity network		
		HRQoL			HRQoL		
		Good	Normal	Poor	Good	Normal	Poor
Male (n)		3,098	1,907	457	3,098	1,907	457
	Angina pectoris^*^	155 (5.0)	131 (6.9)	41 (9.0)	0.09	0.16	0.23
	Arthritis^***^	245 (7.9)	359 (18.8)	131 (28.7)	0.12	0.32	0.55
	Asthma^***^	79 (2.6)	100 (5.2)	41 (9.0)	0.04	0.10	0.22
	Cancer	291 (9.4)	180 (9.4)	46 (10.1)	0.12	0.17	0.19
	CKD^***^	298 (9.6)	263 (13.8)	77 (16.8)	0.18	0.30	0.42
	Depression^***^	40 (1.3)	75 (3.9)	36 (7.9)	0.02	0.08	0.23
	Diabetes^***^	586 (18.9)	429 (22.5)	139 (30.4)	0.31	0.47	0.70
	Hyperlipidemia	558 (18.0)	357 (18.7)	88 (19.3)	0.33	0.42	0.54
	Hypertension^***^	1,440 (46.5)	1,027 (53.9)	255 (55.8)	0.55	0.81	1.11
	Liver disease	77 (2.5)	50 (2.6)	20 (4.4)	0.04	0.05	0.14
	Myocardial infarction^*^	103 (3.3)	79 (4.1)	32 (7.0)	0.07	0.10	0.20
	Renal failure	19 (0.6)	16 (0.8)	7 (1.5)	0.01	0.03	0.05
	Stroke^***^	126 (4.1)	164 (8.6)	98 (21.4)	0.08	0.18	0.48
	Thyroid	46 (1.5)	27 (1.4)	4 (0.9)	0.02	0.03	0.02
	Tuberculosis	289 (9.3)	196 (10.3)	50 (10.9)	0.12	0.18	0.23
Female (n)		2,579	3,270	1,346	2,579	3,270	1,346
	Angina pectoris^***^	97 (3.8)	176 (5.4)	103 (7.7)	0.10	0.15	0.25
	Arthritis^***^	809 (31.4)	1,661 (50.8)	845 (62.8)	0.51	0.90	1.27
	Asthma^***^	108 (4.2)	189 (5.8)	129 (9.6)	0.09	0.14	0.26
	Cancer	128 (5.0)	178 (5.4)	73 (5.4)	0.09	0.11	0.14
	CKD^***^	200 (7.8)	313 (9.6)	174 (12.9)	0.17	0.25	0.39
	Depression^***^	143 (5.5)	300 (9.2)	176 (13.1)	0.12	0.22	0.37
	Diabetes^***^	467 (18.1)	684 (20.9)	324 (24.1)	0.39	0.49	0.68
	Hyperlipidemia^*^	791 (30.7)	1,106 (33.8)	393 (29.2)	0.56	0.76	0.82
	Hypertension^***^	1,390 (53.9)	1,938 (59.3)	842 (62.6)	0.79	1.07	1.36
	Liver disease	39 (1.5)	58 (1.8)	25 (1.9)	0.04	0.04	0.05
	Myocardial infarction^***^	30 (1.2)	56 (1.7)	43 (3.2)	0.04	0.05	0.12
	Renal failure^*^	10 (0.4)	19 (0.6)	16 (1.2)	0.01	0.02	0.04
	Stroke^***^	96 (3.7)	148 (4.5)	132 (9.8)	0.08	0.12	0.29
	Thyroid	145 (5.6)	181 (5.5)	83 (6.2)	0.12	0.13	0.18
	Tuberculosis	122 (4.7)	150 (4.6)	66 (4.9)	0.08	0.11	0.13

HRQoL, health-related quality of life; CKD, chronic kidney disease.

* p<0.05,

*** p<0.001: Significance level for the difference in the prevalence of diseases by HRQoL groups.

**Table 4.** Association rules analysis^1^ of multimorbidity in the poor HRQoL group stratified by sex in older adults (65+)

Disease X		Disease Y	Support (%)	Confidence (%)	Lift
Male (n=457)					
	Hypertension	Diabetes	20.8	37.3	1.2
	Diabetes	Hypertension	20.8	68.3	1.2
	Hypertension	Arthritis	15.8	28.2	1.0
	Arthritis	Hypertension	15.8	55.0	1.0
	Hypertension	Hyperlipidemia	15.1	27.1	1.4
	Hyperlipidemia	Hypertension	15.1	78.4	1.4
	Hypertension	Stroke	13.8	24.7	1.2
	Stroke	Hypertension	13.8	64.3	1.2
	Hypertension	CKD	12.3	22.0	1.3
	CKD	Hypertension	12.3	72.7	1.3
	Stroke	Diabetes	9.4	43.9	1.4
	Diabetes	Stroke	9.4	30.9	1.4
	Hyperlipidemia	Diabetes	8.1	42.0	1.4
	Diabetes	Hyperlipidemia	8.1	26.6	1.4
	Arthritis	Diabetes	6.3	22.1	0.7
	Diabetes	Arthritis	6.3	20.9	0.7
	Diabetes	CKD	6.3	20.9	1.2
	CKD	Diabetes	6.3	37.7	1.2
	Hypertension	Angina pectoris	5.7	10.2	1.1
	Angina pectoris	Hypertension	5.7	63.4	1.1
Female (n=1,346)					
	Hypertension	Arthritis	41.6	66.5	1.1
	Arthritis	Hypertension	41.6	66.3	1.1
	Hypertension	Hyperlipidemia	22.5	36.0	1.2
	Hyperlipidemia	Hypertension	22.5	77.1	1.2
	Hyperlipidemia	Arthritis	20.1	68.7	1.1
	Arthritis	Hyperlipidemia	20.1	32.0	1.1
	Hypertension	Diabetes	19.1	30.5	1.3
	Diabetes	Hypertension	19.1	79.3	1.3
	Arthritis	Diabetes	15.8	25.1	1.0
	Diabetes	Arthritis	15.8	65.4	1.0
	Hyperlipidemia	Diabetes	10.8	36.9	1.5
	Diabetes	Hyperlipidemia	10.8	44.8	1.5
	Hypertension	CKD	10.2	16.3	1.3
	CKD	Hypertension	10.2	78.7	1.3
	Arthritis	Depression	8.8	14.0	1.1
	Depression	Arthritis	8.8	67.0	1.1
	Hypertension	Depression	8.2	13.2	1.0
	Depression	Hypertension	8.2	63.1	1.0
	Hypertension	Stroke	7.8	12.5	1.3
	Stroke	Hypertension	7.8	79.5	1.3

HRQoL, health-related quality of life; CKD, chronic kidney disease.

1 The 20 highest association rules ordered by support value were presented.

## SUPPLEMENTARY MATERIALS

Supplementary Material 1. Study diagram

Supplementary Material 2 Statistical analysis

Supplementary Material 3 Association rules analysis of multimorbidity stratified by HRQoL groups

Supplementary Material 4 Prevalence of diseases and node strength of multimorbidity network stratified by HRQoL groups

Supplementary References
