# Supplementary material for: Multimorbidity patterns by health-related quality of life status in older adults: an association rules and network analysis utilizing the Korea National Health and Nutrition Examination Survey
Source: Epidemiol Health. 2022 Nov 29;44:e2022113. doi: 10.4178/epih.e2022113 (PMC10185967; doi:10.4178/epih.e2022113)
Supplement: Supplementary Material 1. — Study diagram [file epih-44-e2022113-Supplementary-1.docx]

**Multimorbidity patterns by health-related quality of life status in older adults:**

**An association rules and network analysis utilizing Korean National Health and Nutrition Examination Survey**

**SUPPLEMENT MATERIALS**

**Table of contents**

**Supplementary Material 1:** Study diagram

**Supplementary Material 2:** Statistical analysis

- 1. Measurements
  2. Association rules
  3. Network and heatmap analysis

**Supplementary Material 3:** Association rules analysis of multimorbidity stratified by HRQoL groups

**Supplementary Material 4:** Prevalence of diseases and node strength of multimorbidity network stratified by HRQoL groups

**
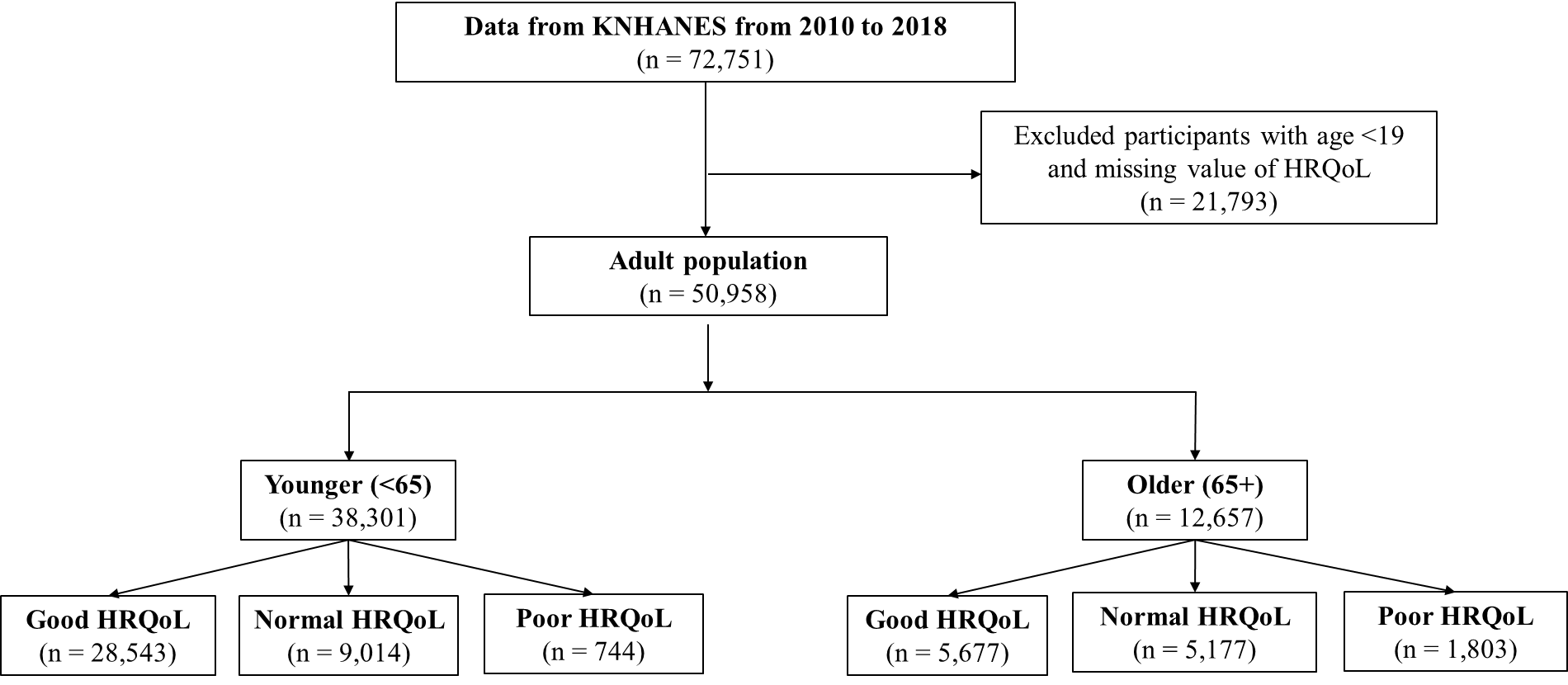
**

**Supplementary Material 1.** Study diagram. Good HRQoL: EQ5D = 1, Normal HRQoL: 0.721 < EQ5D < 1, Poor HRQoL: EQ5D ≤ 0.721
