# Supplementary material for: Multimorbidity patterns by health-related quality of life status in older adults: an association rules and network analysis utilizing the Korea National Health and Nutrition Examination Survey
Source: Epidemiol Health. 2022 Nov 29;44:e2022113. doi: 10.4178/epih.e2022113 (PMC10185967; doi:10.4178/epih.e2022113)
Supplement: Supplementary Material 2 — Statistical analysis [file epih-44-e2022113-Supplementary-2.docx]

**Multimorbidity patterns by health-related quality of life status in older adults:**

**An association rules and network analysis utilizing Korean National Health and Nutrition Examination Survey**

**SUPPLEMENT MATERIALS**

**Table of contents**

**Supplementary Material 1:** Study diagram

**Supplementary Material 2:** Statistical analysis

- 1. Measurements
  2. Association rules
  3. Network and heatmap analysis

**Supplementary Material 3:** Association rules analysis of multimorbidity stratified by HRQoL groups

**Supplementary Material 4:** Prevalence of diseases and node strength of multimorbidity network stratified by HRQoL groups

**Supplementary Material 2:** Statistical analysis

***2.1. Measurements***

The fifteen chronic diseases consistently measured in the three survey periods (V-VII) were selected and used in this study, including hypertension, arthritis, asthma, stroke, angina pectoris, myocardial infarction, diabetes, thyroid, renal failure, hyperlipidemia, liver disease, depression, tuberculosis, chronic kidney disease (CKD), and cancer. All measured cancer types, including stomach, liver, colon, breast, cervix, lung, thyroid cancer, and others, were combined into one variable, “cancer”. The “liver disease” variable was calculated based on hepatitis B, hepatitis C, and liver cirrhosis.

We included age, sex, marital status, income level, working status, and education level as socio-demographic factors in the analysis. *Marital status* was categorised into two groups: married (living with the spouse) and others (single; married, not living together; married, husband/wife passed away; divorced).

Body mass index (BMI), smoking, high-frequency drinking status, walking, sleeping status and stress were included as health determinants factors. *Body mass index (BMI)* was classified as underweight (BMI < 18.5), normal weight (18.5 ≤ BMI < 23.0), overweight (23.0 ≤ BMI < 25.0), and obesity (BMI ≥ 25.0). *Smoking* was measured and coded as "yes" if respondents had smoked 100 cigarettes within one year. *High-frequency drinking status* was defined and coded as "yes" if respondents had drunk more than four times a week, with more than five cups per time (for females) or seven cups per time (for males). *Walking* was defined as "yes" if respondents walked five times a week, with at least 30 minutes per time walking. *Sleeping status* was measured based on the self-reported sleeping hours of respondents and recoded into three categories: short sleeping (<7 hours), optimal sleeping (7-9h), and long sleeping (>9 hours)  [1]. *Stress* was self-reported measurement with binary values (Yes vs. No).

***2.2. Association rules***

Association rules is a popular method for assessing the association between two or more items  [2]. It has been applied to public health and medical fields to uncover the multimorbidity connections in recent years. With the set of two diseases X and Y, the prevalence and relationship of diseases are identified based on three main measurements: support, confidence, and lift, which are commonly used in the previous studies  [3-5]. They are defined as follows:

• Support: The support is defined as the probability of X and Y with the form P(X, Y). It represents the prevalence of co-occurrence of two diseases.

• Confidence: The confidence is the probability of disease Y among people who have the disease X. It is calculated based on the formulation P(Y|X).

• Lift: The lift is measured as $\frac{P(X,Y)}{P\left( X \right) \times P(Y)}$ . This value is also known as the observed/expected ratio and represents the degree of co-occurrence between X and Y. If the lift value equals 1, it means that X and Y are independent, and there is no association between them. Two diseases will have more chance of co-occurrence and significant association if they have a high lift  [3].

*Arules* package in R (version 4.0.4, open-source, available at <https://www.r-project.org>) was used to calculate the association rules with the function *apriori*  [2]. We approached the condition so that only the associations in which two diseases appear are represented.

***2.3. Network and heatmap analysis***

Based on the support value of association rule analysis, we built the network to exceptional estimation and visualisation the interrelationship of multimorbidity in various HRQoL groups. The node strength was calculated based on support value to assess how strongly directed the connection of a node with other nodes in the network  [6, 7]. A network graph consists of nodes (diseases) which are connected by edges (a straight line). The size of the node is proportional to the prevalence of the specific diseases. The color of the nodes is the node strength of a node with other nodes in the network; the darker the color, the higher the value. The thickness of the edge connecting any two diseases in the network is proportional to the number of participants who had both diseases. The method was described in previous studies  [2-5, 8]. Further, to better visualisation, only support values larger than 0.002 were shown. In this part, the weight of the node was included to better evaluate how much important of the disease node. This study utilized the *igraph* package for both network visualisation  [7] and node strength analysis  [6, 9].

The heatmap graph was used to perform all the lift values of association rules through the *ggplot2* package  [2]. The grid points' color indicates the observed to expected ratio. Lift > 1 indicates that two diseases are more likely to co-occur, and higher lift value indicates for higher probability that the two disease co-occurrence.

All statistical analysis was conducted using R statistical software (version 4.0.4, open-source, available at <https://www.r-project.org>).
