# Supplementary material for: Multimorbidity patterns by health-related quality of life status in older adults: an association rules and network analysis utilizing the Korea National Health and Nutrition Examination Survey
Source: Epidemiol Health. 2022 Nov 29;44:e2022113. doi: 10.4178/epih.e2022113 (PMC10185967; doi:10.4178/epih.e2022113)
Supplement: Supplementary Material 3 — Association rules analysis of multimorbidity stratified by HRQoL groups [file epih-44-e2022113-Supplementary-3.docx]

**Multimorbidity patterns by health-related quality of life status in older adults:**

**An association rules and network analysis utilizing Korean National Health and Nutrition Examination Survey**

**SUPPLEMENT MATERIALS**

**Table of contents**

**Supplementary Material 1:** Study diagram

**Supplementary Material 2:** Statistical analysis

- 1. Measurements
  2. Association rules
  3. Network and heatmap analysis

**Supplementary Material 3:** Association rules analysis of multimorbidity stratified by HRQoL groups

**Supplementary Material 4:** Prevalence of diseases and node strength of multimorbidity network stratified by HRQoL groups

**Supplementary Material 3.** Association rules analysis*^a^* of multimorbidity stratified by HRQoL groups in the older adults (65+).

A: Good HRQoL, B: Normal HRQoL, C: Poor HRQoL

| **A. Good HRQoL (n=5,677)** | | | | | | | | | | | |
| --- | --- | --- | --- | --- | --- | --- | --- | --- | --- | --- | --- |
| **Disease X** | |  | | **Disease Y** | | **Support** (%) | **Confidence** (%) | | **Lift** | | |
| Hypertension | |  | | Hyperlipidemia | | 16.4 | 32.8 | | 1.4 | | |
| Hyperlipidemia | |  | | Hypertension | | 16.4 | 68.9 | | 1.4 | | |
| Hypertension | |  | | Diabetes | | 12.4 | 24.8 | | 1.3 | | |
| Diabetes | |  | | Hypertension | | 12.4 | 66.8 | | 1.3 | | |
| Hypertension | |  | | Arthritis | | 10.5 | 21.1 | | 1.1 | | |
| Arthritis | |  | | Hypertension | | 10.5 | 56.6 | | 1.1 | | |
| Hyperlipidemia | |  | | Diabetes | | 6.9 | 29.1 | | 1.6 | | |
| Diabetes | |  | | Hyperlipidemia | | 6.9 | 37.3 | | 1.6 | | |
| Hypertension | |  | | CKD | | 6.3 | 12.7 | | 1.4 | | |
| CKD | |  | | Hypertension | | 6.3 | 71.9 | | 1.4 | | |
| Hyperlipidemia | |  | | Arthritis | | 5.8 | 24.5 | | 1.3 | | |
| Arthritis | |  | | Hyperlipidemia | | 5.8 | 31.3 | | 1.3 | | |
| Arthritis | |  | | Diabetes | | 3.8 | 20.7 | | 1.1 | | |
| Diabetes | |  | | Arthritis | | 3.8 | 20.7 | | 1.1 | | |
| Hypertension | |  | | Cancer | | 3.4 | 6.7 | | 0.9 | | |
| Cancer | |  | | Hypertension | | 3.4 | 45.6 | | 0.9 | | |
| Hypertension | |  | | Tuberculosis | | 3.3 | 6.6 | | 0.9 | | |
| Tuberculosis | |  | | Hypertension | | 3.3 | 45.7 | | 0.9 | | |
| Diabetes | |  | | CKD | | 3.0 | 16.0 | | 1.8 | | |
| CKD | |  | | Diabetes | | 3.0 | 33.9 | | 1.8 | | |
| **B. Normal HRQoL (n=5,177)** | | | | | | | | | | |  |
| **Disease X** |  | | **Disease Y** | | **Support** (%) | | | **Confidence** (%) | | **Lift** |  |
| Hypertension |  | | Arthritis | | 23.4 | | | 40.9 | | 1.0 |  |
| Arthritis |  | | Hypertension | | 23.4 | | | 60.0 | | 1.0 |  |
| Hypertension |  | | Hyperlipidemia | | 20.4 | | | 35.6 | | 1.3 |  |
| Hyperlipidemia |  | | Hypertension | | 20.4 | | | 72.1 | | 1.3 |  |
| Hypertension |  | | Diabetes | | 16.1 | | | 28.1 | | 1.3 |  |
| Diabetes |  | | Hypertension | | 16.1 | | | 74.9 | | 1.3 |  |
| Hyperlipidemia |  | | Arthritis | | 13.8 | | | 48.7 | | 1.2 |  |
| Arthritis |  | | Hyperlipidemia | | 13.8 | | | 35.3 | | 1.2 |  |
| Hyperlipidemia |  | | Diabetes | | 9.1 | | | 32.3 | | 1.5 |  |
| Diabetes |  | | Hyperlipidemia | | 9.1 | | | 42.4 | | 1.5 |  |
| Hypertension |  | | CKD | | 8.4 | | | 14.6 | | 1.3 |  |
| CKD |  | | Hypertension | | 8.4 | | | 75.2 | | 1.3 |  |
| Arthritis |  | | Diabetes | | 8.1 | | | 20.6 | | 1.0 |  |
| Diabetes |  | | Arthritis | | 8.1 | | | 37.5 | | 1.0 |  |
| Hypertension |  | | Stroke | | 4.6 | | | 8.0 | | 1.3 |  |
| Stroke |  | | Hypertension | | 4.6 | | | 76.0 | | 1.3 |  |
| Hypertension |  | | Depression | | 4.1 | | | 7.2 | | 1.0 |  |
| Depression |  | | Hypertension | | 4.1 | | | 56.8 | | 1.0 |  |
| Hypertension |  | | Angina pectoris | | 4.1 | | | 7.1 | | 1.2 |  |
| Angina pectoris |  | | Hypertension | | 4.1 | | | 68.7 | | 1.2 |  |

| **C. Poor HRQoL (n=1,803)** | | | | | |
| --- | --- | --- | --- | --- | --- |
| **Disease X** |  | **Disease Y** | **Support** (%) | **Confidence** (%) | **Lift** |
| Hypertension |  | Arthritis | 35.1 | 57.6 | 1.1 |
| Arthritis |  | Hypertension | 35.1 | 64.8 | 1.1 |
| Hypertension |  | Hyperlipidemia | 20.6 | 33.9 | 1.3 |
| Hyperlipidemia |  | Hypertension | 20.6 | 77.3 | 1.3 |
| Hypertension |  | Diabetes | 19.5 | 32.1 | 1.2 |
| Diabetes |  | Hypertension | 19.5 | 76.0 | 1.2 |
| Hyperlipidemia |  | Arthritis | 16.4 | 61.3 | 1.1 |
| Arthritis |  | Hyperlipidemia | 16.4 | 30.2 | 1.1 |
| Arthritis |  | Diabetes | 13.4 | 24.7 | 1.0 |
| Diabetes |  | Arthritis | 13.4 | 52.1 | 1.0 |
| Hypertension |  | CKD | 10.7 | 17.6 | 1.3 |
| CKD |  | Hypertension | 10.7 | 76.9 | 1.3 |
| Hyperlipidemia |  | Diabetes | 10.1 | 37.8 | 1.5 |
| Diabetes |  | Hyperlipidemia | 10.1 | 39.3 | 1.5 |
| Hypertension |  | Stroke | 9.3 | 15.3 | 1.2 |
| Stroke |  | Hypertension | 9.3 | 73.0 | 1.2 |
| Hypertension |  | Depression | 7.4 | 12.1 | 1.0 |
| Depression |  | Hypertension | 7.4 | 62.7 | 1.0 |
| Arthritis |  | Depression | 7.3 | 13.5 | 1.2 |
| Depression |  | Arthritis | 7.3 | 62.3 | 1.2 |

*Notes: HRQoL = health-related quality of life; CKD = chronic kidney disease.*

*^a^The 20 highest association rules ordered by support value were presented.*
