# Supplementary material for: Multimorbidity patterns by health-related quality of life status in older adults: an association rules and network analysis utilizing the Korea National Health and Nutrition Examination Survey
Source: Epidemiol Health. 2022 Nov 29;44:e2022113. doi: 10.4178/epih.e2022113 (PMC10185967; doi:10.4178/epih.e2022113)
Supplement: Supplementary Material 4 — Prevalence of diseases and node strength of multimorbidity network stratified by HRQoL groups [file epih-44-e2022113-Supplementary-4.docx]

**Multimorbidity patterns by health-related quality of life status in older adults:**

**An association rules and network analysis utilizing Korean National Health and Nutrition Examination Survey**

**SUPPLEMENT MATERIALS**

**Table of contents**

**Supplementary Material 1:** Study diagram

**Supplementary Material 2:** Statistical analysis

- 1. Measurements
  2. Association rules
  3. Network and heatmap analysis

**Supplementary Material 3:** Association rules analysis of multimorbidity stratified by HRQoL groups

**Supplementary Material 4:** Prevalence of diseases and node strength of multimorbidity network stratified by HRQoL groups

**Supplementary Material 4.** Prevalence of diseases and node strength of multimorbidity network stratified by HRQoL groups in the older adults (65+).

| **Type of diseases** | **Prevalence of diseases** | | | |  | **Node strength of multimorbidity network** | | |  |
| --- | --- | --- | --- | --- | --- | --- | --- | --- | --- |
|  | **Total**  **n=12,657 (%)** | **Good HRQoL**  **n=5,677 (%)** | **Normal HRQoL**  **n=5,177 (%)** | **Poor HRQoL**  **n=1,803 (%)** |  | **Good HRQoL**  **n=5,677** | **Normal HRQoL**  **n=5,177** | **Poor HRQoL**  **n=1,803** | |
| Angina pectoris^**^ | 703 (5.6) | 252 (4.4) | 307 (5.9) | 144 (8.0) |  | 0.09 | 0.15 | 0.25 | |
| Arthritis^**^ | 4050 (32.0) | 1054 (18.6) | 2020 (39.0) | 976 (54.1) |  | 0.30 | 0.69 | 1.09 | |
| Asthma^**^ | 646 (5.1) | 187 (3.3) | 289 (5.6) | 170 (9.4) |  | 0.06 | 0.13 | 0.25 | |
| Cancer | 896 (7.1) | 419 (7.4) | 358 (6.9) | 119 (6.6) |  | 0.11 | 0.13 | 0.16 | |
| CKD^**^ | 1325 (10.5) | 498 (8.8) | 576 (11.1) | 251 (13.9) |  | 0.18 | 0.27 | 0.40 | |
| Depression^**^ | 770 (6.1) | 183 (3.2) | 375 (7.2) | 212 (11.8) |  | 0.06 | 0.17 | 0.34 | |
| Diabetes^**^ | 2629 (20.8) | 1053 (18.5) | 1113 (21.5) | 463 (25.7) |  | 0.35 | 0.48 | 0.69 | |
| Hyperlipidemia^**^ | 3293 (26.0) | 1349 (23.8) | 1463 (28.3) | 481 (26.7) |  | 0.43 | 0.64 | 0.75 | |
| Hypertension^**^ | 6892 (54.5) | 2830 (49.9) | 2965 (57.3) | 1097 (60.8) |  | 0.66 | 0.98 | 1.30 | |
| Liver disease | 269 (2.1) | 116 (2.0) | 108 (2.1) | 45 (2.5) |  | 0.04 | 0.04 | 0.07 | |
| Myocardial infarction^**^ | 343 (2.7) | 133 (2.3) | 135 (2.6) | 75 (4.2) |  | 0.05 | 0.07 | 0.14 | |
| Renal failure^*^ | 87 (0.7) | 29 (0.5) | 35 (0.7) | 23 (1.3) |  | 0.01 | 0.02 | 0.04 | |
| Stroke^**^ | 764 (6.0) | 222 (3.9) | 312 (6.0) | 230 (12.8) |  | 0.08 | 0.14 | 0.34 | |
| Thyroid^*^ | 486 (3.8) | 191 (3.4) | 208 (4.0) | 87 (4.8) |  | 0.06 | 0.09 | 0.14 | |
| Tuberculosis | 873 (6.9) | 411 (7.2) | 346 (6.7) | 116 (6.4) |  | 0.10 | 0.13 | 0.15 | |

*Notes: HRQoL = health-related quality of life; CKD = chronic kidney disease.*

*Significant difference level: *p<.05; **p<.001*
