# Supplementary material for: Multimorbidity patterns by health-related quality of life status in older adults: an association rules and network analysis utilizing the Korea National Health and Nutrition Examination Survey
Source: Epidemiol Health. 2022 Nov 29;44:e2022113. doi: 10.4178/epih.e2022113 (PMC10185967; doi:10.4178/epih.e2022113)
Supplement: Supplementary References [file epih-44-e2022113-Supplementary-References.docx]

**Multimorbidity patterns by health-related quality of life status in older adults:**

**An association rules and network analysis utilizing Korean National Health and Nutrition Examination Survey**

**SUPPLEMENT MATERIALS**

**Table of contents**

**Supplementary Material 1:** Study diagram

**Supplementary Material 2:** Statistical analysis

- 1. Measurements
  2. Association rules
  3. Network and heatmap analysis

**Supplementary Material 3:** Association rules analysis of multimorbidity stratified by HRQoL groups

**Supplementary Material 4:** Prevalence of diseases and node strength of multimorbidity network stratified by HRQoL groups

**References**

1.Kim SY LM, Lim WJ, Kim SI, Lee YJ. Associations of 25-Hydroxyvitamin D Levels and Arthritis with Sleep Duration: The Korean National Health and Nutrition Examination Survey 2008–2014. Nature and Science of Sleep 2020;12:883–894.

2.Michael Hahsler BG, Kurt Hornik. arules – A Computational Environment for Mining Association Rules and Frequent Item Sets. Journal of Statistical Software 2005;14

3.Yoonju Lee HK, Hyesun Jeong, Yunhwan Noh Patterns of Multimorbidity in Adults: An Association Rules Analysis Using the Korea Health Panel. International Journal of Environmental Research and Public Health 2020;17

4.Hernández B, Reilly, Richard B., Kenny, Rose Anne. Investigation of multimorbidity and prevalent disease combinations in older Irish adults using network analysis and association rules. Scientific Reports 2019;9:145-167.

5.Fabian P. Held FB, Danijela Gnjidic, Vasant Hirani, Vasikaran Naganathan, Louise M. Waite, Markus J. Seibel, Jennifer Rollo, David J. Handelsman, Robert G. Cumming, David G. Le Couteur. Association Rules Analysis of Comorbidity and Multimorbidity: The Concord Health and Aging in Men Project. Journals of Gerontology: Medical Sciences 2016;71:625-631.

6.Hevey D. Network analysis: a brief overview and tutorial. Health Psychology and Behavioral Medicine 2018;6:301-328.

7.Ognyanova K. Network visualization with R. Available from:

8.Jin Hee Kim KYS, Dong Wook Shin, Sang Hyuk Kim, Jae Won Yun, Jung Hyun Shin, Mi So Kang, Eui Heon Chung, Kyoung Hun Yoo, Jae Moon Yun. Network analysis of human diseases using Korean nationwide claims data. Journal of Biomedical Informatics 2016;61:276–282.

9.Jonas Dalege DB, Frenk van Harreveld, and Han L. J. van der Maas. Network Analysis on Attitudes: A Brief Tutorial. Social Psychological and Personality Science 2017;8:528-537.
